# Big Biking Payoff: Alternative Transportation Could Net Midwest over $8 Billion

**DOI:** 10.1289/ehp.120-a34b

**Published:** 2012-01-01

**Authors:** Kellyn S. Betts

**Affiliations:** Kellyn S. Betts has written about environmental contaminants, hazards, and technology for solving environmental problems for publications including *EHP* and *Environmental Science & Technology* for more than a dozen years.

A New Year’s resolution to bike rather than drive for short trips could improve both personal health and regional air quality. A team led by researchers from the University of Wisconsin at Madison is the first to quantify these benefits in the United States to show they may have a significant economic payoff [*EHP* 120(1):68–76; Grabow et al.].

In 2010 another team concluded that the health benefits of biking substantially outweigh the risks posed by accidents and exposure to pollution based on a literature review comparing the use of cars and bikes for trips under 5 miles [*EHP* 118(8):1109–1116]. The new study expands that concept by combining data on transportation, automotive emissions, and health effects of exposure to air pollution to estimate how switching to biking for round trips under 5 miles could impact both air quality and health care costs. Short trips contribute disproportionately to air pollution because a large fraction of toxic automotive emissions, including 25% of the volatile organic compounds (VOCs) and 19% of the primary fine particulate matter (PM_2.5_), are generated in the first few miles of travel before pollution control devices have reached their operating temperatures.

**Figure:**
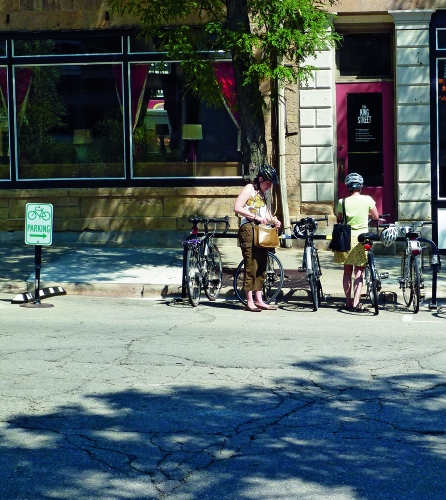
Using a bike for short trips may offer health benefits at multiple levels. © 2012 Arthur Ross

The new study focused on 11 urban and suburban Metropolitan Statistical Areas in Illinois, Indiana, Ohio, Michigan, Minnesota, and Wisconsin. The model for air-quality improvement assumed that car use for all trips of 5 miles or less round trip from April through October were replaced by an alternative means of transportation such as walking, biking, or mass transit. The model for health benefits assumed that 50% of these short trips were taken by bicycle.

Under these conditions the authors project that annual average urban PM_2.5_ concentrations would decline by 0.1 µg/m^3^. Estimating effects of vehicle emissions on ground-level ozone is less straightforward; because VOCs can limit the production of ozone in urban areas, reducing automotive emissions (and thus VOC concentrations) would slightly increase ozone in most cities. But the nonlinear interplay of emissions and meteorology in atmospheric chemistry and transport means reductions in automotive VOCs and nitrogen oxides would still reduce ozone at a regional level.

The models pegged the net health benefit from improved air quality—a benefit that extends beyond city limits—at $4.94 billion per year. The $3.8 billion in estimated annual health benefits that urbanites would accrue from biking were the result of avoided mortality and reduced health care costs through increased physical activity.

Strengths of the study include its cross-disciplinary focus and use of up-to-date models incorporating highly localized emissions and travel data. Although the study acknowledges the health costs associated with car and bike crashes as well as the benefits of increased walking to public transportation, it does not attempt to quantify them.

